# Prevalence and prognosis of *PIK3CA* mutations in Bulgarian patients with metastatic breast cancer receiving endocrine therapy in first‐line setting

**DOI:** 10.1002/cnr2.1966

**Published:** 2023-12-26

**Authors:** R. Gencheva, M. Petrova, P. Kraleva, S. Hadjidekova, M. Radanova, N. Conev, D. Stoyanov, J. Arabadjiev, E. Tazimova, S. Bachurska, M. Eneva, M. Tsvetkova, G. Zhbantov, T. Karanikolova, D. Manov, A. Ivanova, M. Taushanova‐Hadjieva, R. Staneva, E. Dimitrova, I. Donev

**Affiliations:** ^1^ Clinic of Medical Oncology MHAT “Nadezhda” Sofia Bulgaria; ^2^ Department of Medical Genetics, Medical Faculty Medical University of Sofia Sofia Bulgaria; ^3^ Department of Biochemistry, Molecular Medicine and Nutrigenomics Medical University of Varna Varna Bulgaria; ^4^ Clinic of Medical Oncology University Hospital “St. Marina” Varna Bulgaria; ^5^ Clinic of Medical Oncology University Hospital Acibadem City Clinic Tokuda Sofia Bulgaria; ^6^ Department of General and Clinicalpathology University Specialised Hospital for Oncology Sofia Bulgaria; ^7^ Department of Hospital Pharmacy “Nadezhda” Sofia Bulgaria; ^8^ Medical Affairs Novartis Bulgaria Sofia Bulgaria

**Keywords:** breast cancer, clinical outcome, *PIK3CA* (phosphatidylinosiol 3 kinase)‐mutation

## Abstract

**Background and aims:**

In approximately 40% of patients with HER2‐negative/HR‐positive breast cancer tumors, the *PIK3CA* gene is mutated. Despite this, clinical outcomes vary between studies in this cohort. We aimed to ascertain the prevalence of *PIK3CA* mutations in patients with metastatic HR+/HER2– breast in Bulgaria, as well the evaluation and comparison of progression free survival (PFS) between wild‐type (WT) and mutation‐positive groups in the real‐world setting.

**Methods:**

Three oncology centers in Bulgaria collected 250 tissue samples between 2016 and 2022 for this multicentric retrospective study. *PIK3CA* mutations were identified using Real‐Time qPCR. The median follow‐up period was 35 months.

**Results:**

The mean age of the mutant cohort was 57.6 ± 11.6 years, compared to 56.5 ± 12.2 years for the WT cohort (*p* = .52). The percentage of patients with visceral metastasis was 58.8% (*n* = 147). Approximately 84.3% (*n* = 210) of the patients had reached postmenopause. 29.2% (*n* = 73) of the patients had *PIK3CA* mutations. The predominant mutation was present in exon 20, H1047R (46.5%). We found a significant correlation only between the presence of a mutation and the metastatic diseases at diagnosis (*p* = .002). As first‐line therapy, 67.1% of patients received endocrine therapy (ET) plus cyclin dependent kinase (CDK4/6) inhibitor, while the remainder received ET alone. The median PFS of patients in the group with the mutation was 32 months (95%, CI: 22–40) compared to 24 months in the WT cohort ((95%, CI: 21–36) (*p* = .45)); HR = 0.86 (95%, CI: 0.5–1.3) (*p* = .46). We corroborated our conclusion using propensity matching score analysis, (36 months [95% CI: 20–40] vs. 26 months [95% CI: 21–38], [*p* = .69]).

**Conclusions:**

We found that the prevalence of *PIK3CA* mutations in our patients was comparable to what has been reported in other nations. Our results suggest that *PIK3CA* mutational status has no bearing to ET efficacy in first‐line setting.

## 1 INTRODUCTION

Breast cancer (BC) is a socially significant disease since it is the most prevalent neoplasm affecting women in Europe^1^ and the most prevalent non‐cutaneous cancer among women in the United States.^2^ More than 70% of BC cases are positive for hormone receptors (HR+) and negative for human epidermal growth factor receptor 2 (HER2–).^3^, ^4^ The frequency of *PIK3CA* mutations (*PIK3CA*muts) varies depending on the molecular characteristic of BC.^5^ *PIK3CA*muts are the most prevalent mutations within this HR+/HER2– subgroup, with an estimated prevalence of 30%–40%.^6^, ^7^ Intracellularly, phosphatidylinositol‐3‐kinase (PI3K) mediates several processes, such as enhancing cell transformation, tumor initiation and proliferation, and apoptosis resistance.^8^, ^9^, ^10^ Its action is driven by hormones and extracellular growth factors.^10^ Based on their primary structure and in vitro substrate, PI3Ks are classified into three classes. The most researched class is I, which is subdivided into IA and IB based on its interaction with several regulatory subunits and upstream activators.^8^ Class IA PI3K is made up of a regulatory subunit called P85 and a catalytic subunit called P110. P110 is available in three isoforms: p110, p110, and p110.^9^, ^11^

The clinical results of patients with *PIK3CA*muts vary among studies. A meta‐analysis has shown that the presence of *PIK3CA*muts is a negative prognostic factor associated with significantly shorter progression‐free survival (PFS) by about 2 months and shorter overall survival (OS) by about 8 months.^12^ The studies included in the meta‐analysis were heterogeneous, and trials that did not target *PIK3CA*muts had not reported stratified baseline characteristics by *PIK3CA*muts status.^12^ It is largely unknown whether novel therapies, such as cyclin dependent kinase (CDK4/6) inhibitors, may alter the prognosis for patients with *PI3KCA*muts.

This observational study aimed to ascertain the prevalence of *PIK3CA* mutations in patients with metastatic HR+/HER2– BC in Bulgaria, as well the evaluation and comparison of PFS between WT and mutant cohorts in the real world.

## 2 MATERIALS AND METHODS

### 2.1 Patients selection end study endpoints

This study investigated 250 patients with metastatic HR+/HER2–BC treated between January 2016 and November 2022 at three hospitals in Bulgaria (Hospital “Nadezhda,” Sofia, Hospital “Tokuda,” Sofia, University Hospital “St. Marina,” Varna). The hospital's Scientific Research Ethics Committee gave their approval for the study. Before enrolling in the trial, all patients gave tumor samples and granted informed consent (Version 1/02.Apr.2021). This research was carried out in compliance with the Declaration of Helsinki. In compliance with stringent confidentiality rules, demographic and clinicopathological data were collected from computerized medical records.

The study included patients with HR+/HER2– advanced BC, either newly diagnosed or with recurrent locoregional BC, not amenable to surgical resection or radiation with curative aim or metastatic disease. Eligible patients were ≥18 years old, of either menopausal status (premenopausal or postmenopausal), with histologically confirmed BC. Fifteen patients had chemotherapy as first line of treatment, the remaining patients all underwent first‐line endocrine therapy. There were six patients with a positive *PIK3CA*‐ mutation and nine without a mutation. Regarding the duration and effect of the chemotherapy, no data is available. As they progressed, they received a first‐line of hormone therapy. The primary endpoint of the study was to establish the prevalence of *PIK3CA*muts in Bulgarian patients with metastatic HR+/HER2–BC. The secondary endpoints were to evaluate and compare PFS on first‐line endocrine therapy and OS between WT and mutant cohorts in the real world. For one WT patient we missed data for survival analysis. The clinical response of tumors was assessed every two to 4 months using the Response Assessment Criteria in Solid Tumors (version 1.1). Computed tomography was used for staging the patients prior to treatment. Time elapsed between therapy initiation and tumor progression or death from any cause was defined as PFS. During of the first‐line endocrine therapy, 12 patients died. While six had a mutation, the remaining six were negative. We lack information about the cause of death of these patients.

In order to evaluate the differential impact of first‐line therapy decisions on outcome metrics the cohort was sampled and balanced using propensity score matching (PSM), taking into account factors such as first‐line endocrine therapy (ET), menopausal status, and a site of metastatic disease. The matching method was 2:1 closest neighbor with 20% caliber.

### 2.2 Sample processing and analysis

After identifying areas with a high concentration of tumors using hematoxylin and eosin (H&E) staining, DNA was isolated from formalin‐fixed paraffin‐embedded (FFPE) tissue that had been microdissected. This was done using the QIAamp® DSP DNA FFPE tissue kit. A Real‐Time qPCR assay was employed to detect 11 mutations in the *PIK3CA* gene, specifically in exons 7, 9, and 20. The mutations screened for were p.C420R (exon7), p.E542K, p.E545A, p.E545D [c.1635G > T only], p.E545G, p.E545K, p.Q546E, and p.Q546R (exon 9) and p.H1047L, p.H1047R, and p.H1047Y (exon 20). The Rotor‐Gene Q MDx 5plex HRM instrument was used to automate the amplification and detection process.

### 2.3 Statistical analysis

Data was managed and analyzed using SPSS version 23 and EZR. Demographic data were presented using various statistical measures, including frequencies, percentages, medians, means, and standard deviations. The associations between the existence of *PIK3CA* mutations and various clinicopathological parameters of patients were assessed using the Mann–Whitney *U* test and the *χ* ^2^ test. Survival curves were constructed using the Kaplan–Meier method, and differences were evaluated using the log‐rank test. Hazard ratios and 95% confidence intervals were calculated using the Cox proportional‐hazards regression model. A *p*‐value of less than .05 was accepted as statistically significant.

## 3 RESULTS

### 3.1 Baseline clinical characteristics

From 2016till2022, the trial included 250 patients with metastatic HR+/HER2–BC. Table 1 summarizes the relationship between the major clinical characteristics and *PIK3CA*muts status. The mean age was 57.6 ± 11.6 years in patients with *PIK3CA*muts and 56.5 ± 12.6 years in WT group (*p* = .522). Only one male patient was included in our study. 15.7% (*N* = 39) of the population were premenopausal, whereas 84.3% (*N* = 210) were postmenopausal. Those with visceral disease (58.5%, *N* = 147) and those with bone‐only disease (41.2%, *N* = 103) were categorized into two primary groups based on the nature of metastases.

**TABLE 1:** Relationship between baseline clinicopathological characteristics of patients and the *PIK3CA*‐mutation status.

	*PIK3CA* positive (*n* = 73)	*PIK3CA* negative (*n* = 177)	*p*‐value
Age (mean ± SD)	57.6 ± 11.6	56.5 ± 12.6	.522
*Menopausal status*			.915
Premenopausal, *n* (%)	11 (15.3)	28 (15.8)	
Postmenopausal, *n* (%)	61 (84.7)	149 (84.2)	
*Metastatic sites at the time of diagnosis of metastatic BC*			.794
Visceral, *n* (%)	42 (57.5)	105 (59.3)	
Bone‐only, *n* (%)	31 (42.5)	72 (40.7)	
Stage at the initial diagnosis of a breast cancer			.003
Metastatic, *n* (%)	45 (61.6)	72 (40.7)	
Nonmetastatic, *n* (%)	28 (38.4)	105 (59.3)	
*Sample used for detection of PIK3CA mutation*			.496
Archival tissue, *n* (%)	57 (78.1)	145 (81.9)	
Most recent biopsy tissue, *n* (%)	16 (21.9)	32 (18.1)	
*CT as adjuvant/neoadjuvant therapy*			.034
Yes, *n* (%)	24 (32.9)	84 (47.5)	
No, *n* (%)	49 (67.1)	93 (52.5)	
*Type of adjuvant ET*			.491
Tamoxifen, *n* (%)	7 (25.9)	35 (37.2)	
AI, *n* (%)	18 (66.7)	55 (58.5)	
Tamoxifen + switchtoAI, *n* (%)	2 (7.4)	4 (4.3)	
*Duration of adjuvant ET*			.013
Primary endocrine resistance, *n* (%) (progression less than 2 years since the beginning of ET)	8 (12.3)	34 (23.1)	
Secondary endocrine resistance, *n* (%) (progression between 2 and 5 years since the beginning of ET)	5 (7.7)	28 (19)	
Secondary endocrine resistance, *n* (%) (progression less than 1 year after the end of adjuvant therapy)	2 (3.1)	9 (6.1)	
More than 1 year after the end of adjuvant therapy, *n* (%)	12 (18.5)	23 (15.6)	

Abbreviations: AI, aromatase inhibitor; BC, breast cancer; CDK4/6, cyclin‐dependent kinase 4/6 inhibitor; CT, chemotherapy; ET, endocrine therapy.

### 3.2 *PI3KCA* mutation prevalence

**FIGURE 1:**
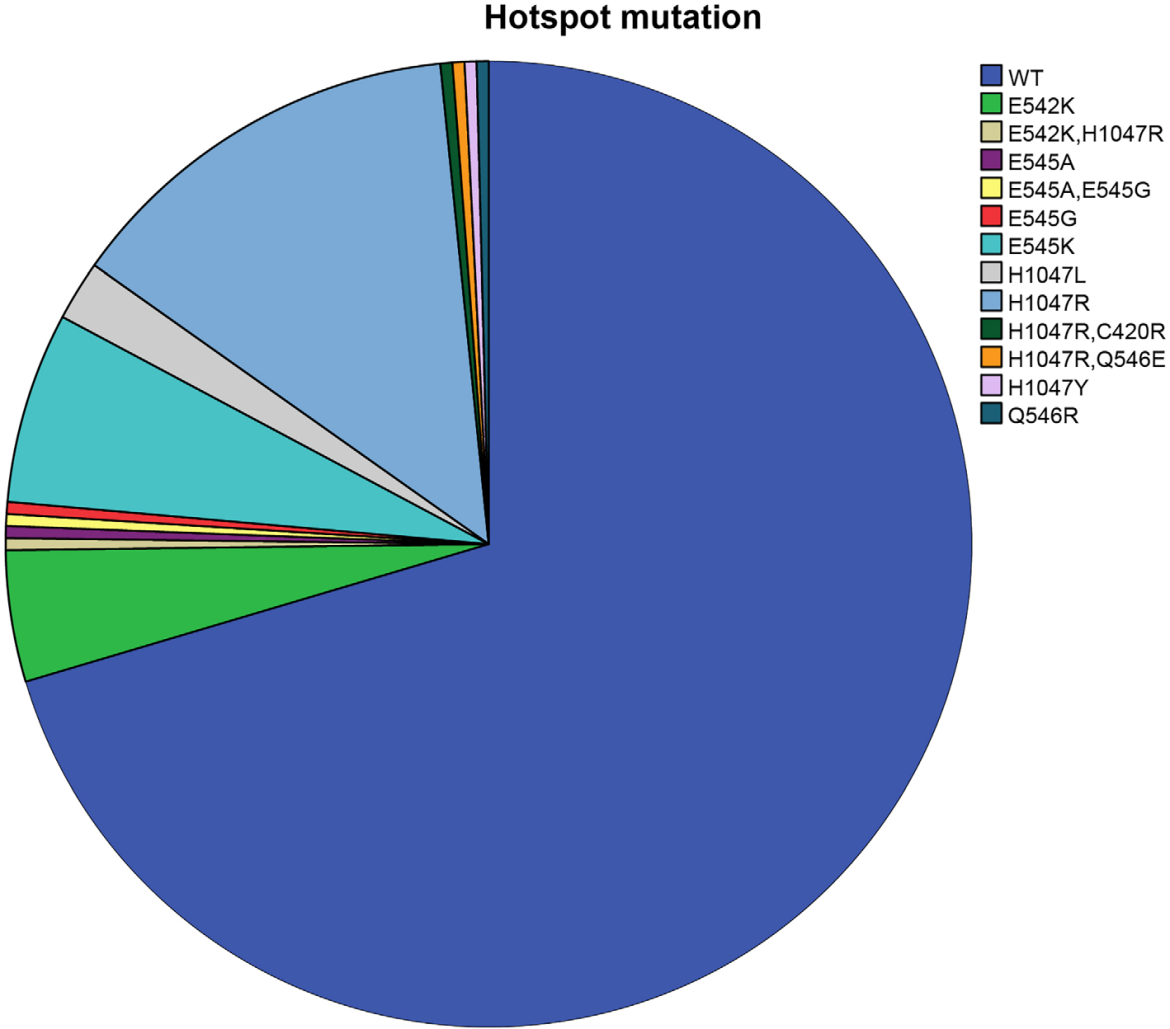
Prevalence and types of *PIK3CA* mutations in Bulgaria. The presence of *PIK3CA* mutations was identified in 29.2% (*N* = 73) of the analyzed patients. As determined by localization, the distribution of mutations was as follows: 39.7% were found in exon 9, 54.8% in exon 20, and 5.5% simultaneously in both exons. The exon 20 mutation with the highest frequency was p.H1047R (46.5%).

Presence of *PIK3CA*muts was identified in 29.2% (*N* = 73) of the patients analyzed, where as it was absent in 70.8% (*N* = 177) of the patients. As determined by localization, the distribution of mutations was as follows: 39.7% were discovered in exon9, 54.8% in exon20, and 5.5% simultaneously in both exons. The exon 20 mutation with the highest frequency was H1047R (46.5%) (Figure 1).

We compared *PIK3CA*muts incidence in our study with that of TCGA (The Cancer Genome Atlas‐USA) as of January 4, 2023. The TCGA cancer genomics data is a publicly accessible database. We extracted information concerning 933 patients. Only patients with metastatic breast cancer, HR(+) HER2(−) status were included in the sample. Our study found that there was no significant difference in the prevalence of mutations between the Bulgarian and American populations (*p* = .10).

### 3.3 Association between *PIK3CA*muts status and baseline clinicopatholo‐gical features of the patients

Patients with *PIK3CA*muts were 84.7% (*N* = 61) postmenopausal and 15.3% (*N* = 11) premenopausal. In the WT group, the distribution was the same: postmenopausal—84.2% (*N* = 149) and premenopausal—15.8% (N = 28). In the *PIK3CA*mut group—57.5% (*N* = 42) of the patientshad visceral disease and 42.5% (*N* = 31)—bone‐only disease. In the WT cohort −59.7% (*N* = 105) of the patients had visceral disease and 40.3% (*N* = 72) had bone‐only disease.

We noticed that only the existence of *PIK3CA*muts was significantly associated with presence of metastatic disease when diagnosed (*p* = .002), usage of chemotherapy in neo/adjuvant contexts (*p* = .034), and presence of endocrine resistance in adjuvant settings (*p* = .013) among all clinicopathological parameters of the patients (Table 1).

### 3.4 Association between *PIK3CA*muts status and clinical outcome

**FIGURE 2:**
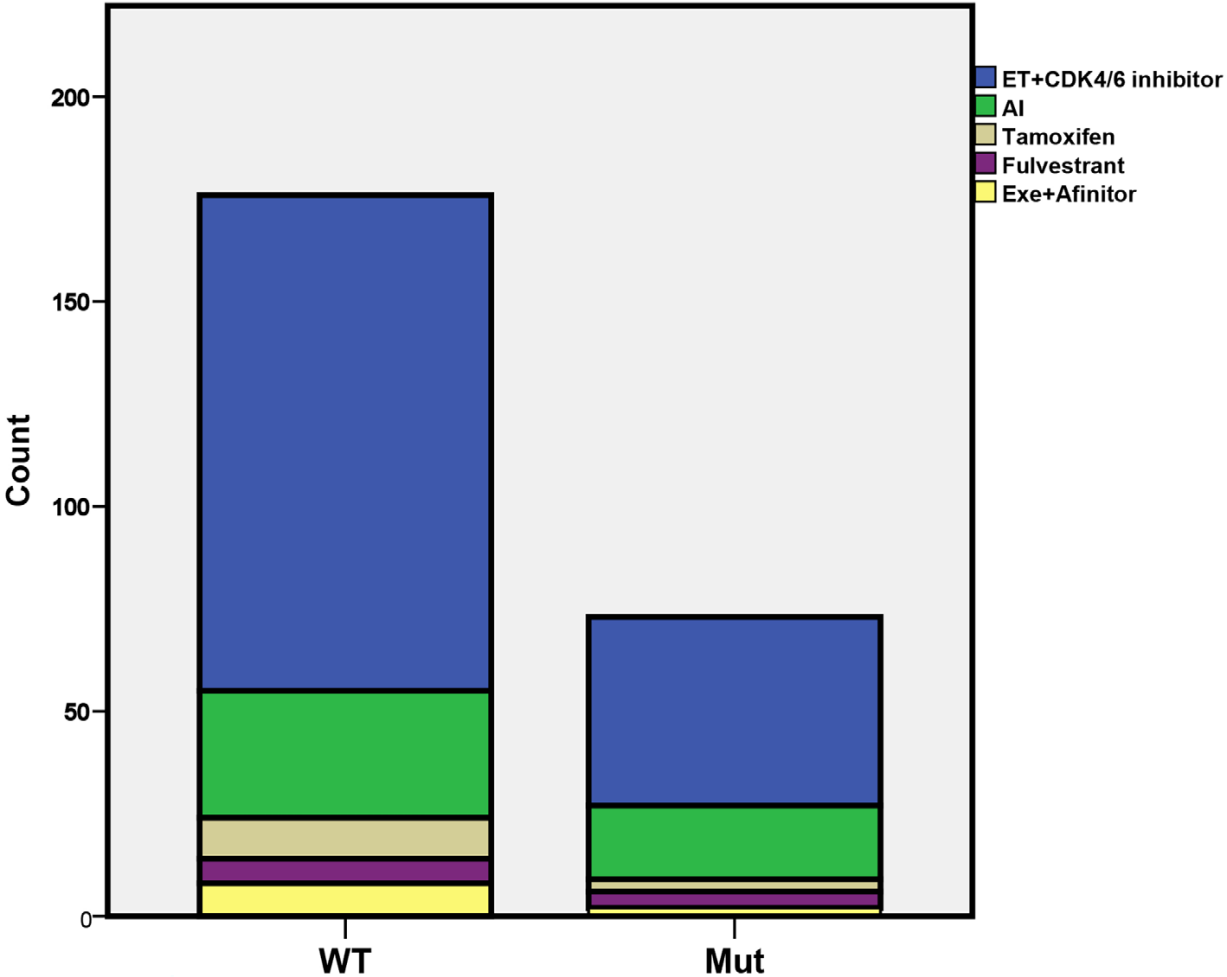
Types of endocrine therapy used as a first line in Bulgaria. There was not a significant difference in the types of endocrine therapy used as a first‐line treatment between patients with *PIK3CA* mutations and WT cohorts (*χ* ^2^ = 2.76, *p* = .55). AI, aromatase inhibitor; CDK4/6, cyclin‐dependent kinase 4/6 inhibitor; ET, endocrine therapy; Exe, exemestane.

**FIGURE 3:**
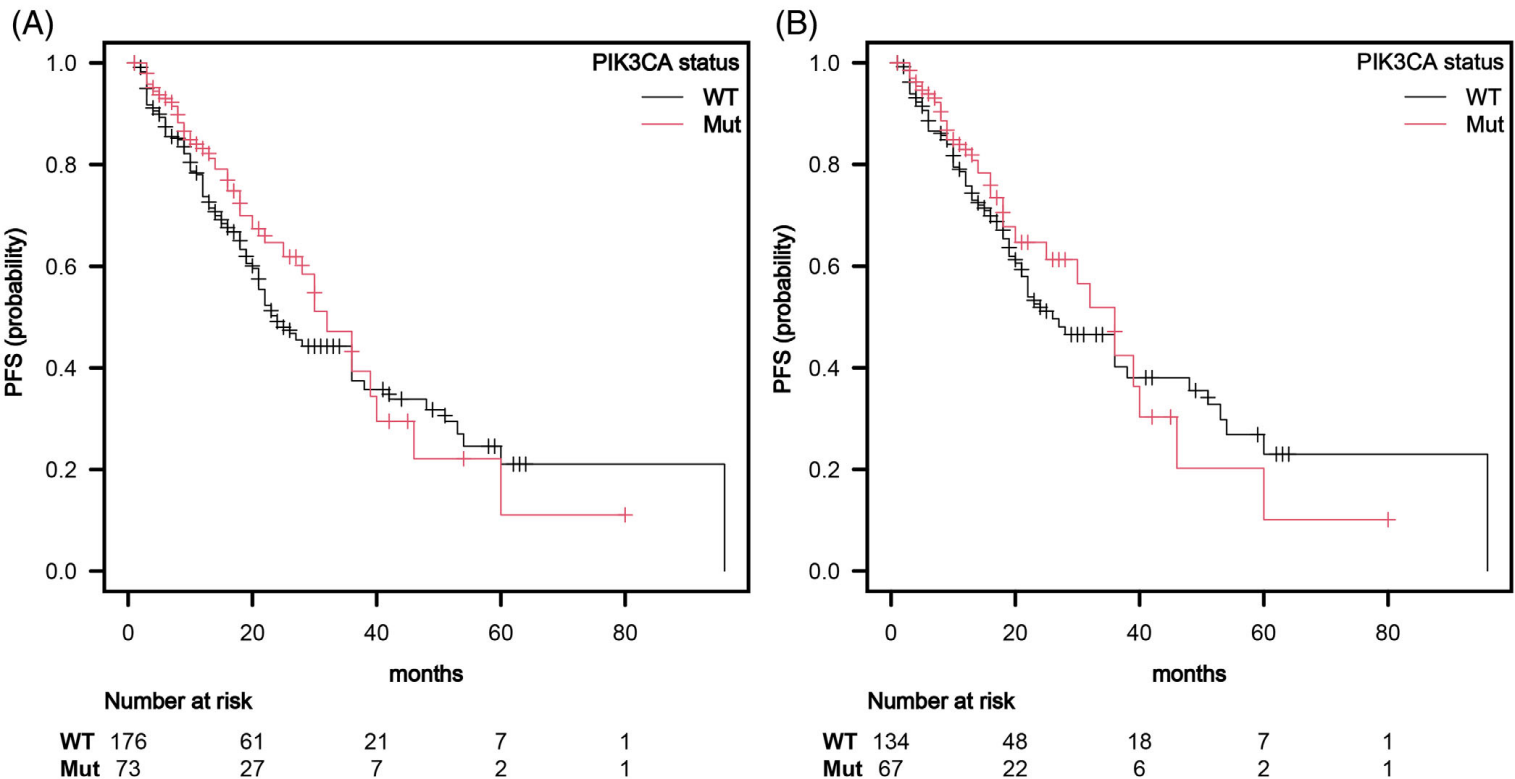
Kaplan‐Maier estimates of progression free survival (PFS). (A). Patients with *PIK3CA* mutations did not have significantly different median PFS compared to WT patients (32 months [95%, CI: 22–40] vs. 24 months [95%, CI: 21–36], [*p* = .45]). (B). In the propensity matching score analysis (matching data for treatment administered as a first‐line endocrine therapy (ET), menopausal status, and locations of metastatic disease), patients with *PIK3CA* mutations did not have significantly different median PFS compared to WT (36 months [95%, CI: 20–40] vs. 26 months [95%, CI: 21–38], [*p* = .69]).

As first‐line treatment 67% of patients underwent ET plus CDK4/6 inhibitor, while the remaining patients had ET alone. There was no notable difference in the types of first‐line endocrine therapy used between the two groups, as shown in Figure 2. The median PFS of patients with *PIK3CA*muts was not significantly longer than that of patients without mutations. The PFS of patients in the group with the mutation was 32 months (95%, CI: 22–40) compared to 24 months in WT cohort (95%, CI: 21–36, *p* = .45; HR = 0.86 (95%, CI: 0.5–1.3, *p* = .46), Figure 3A). Using propensity matching score analysis (matching data for treatment received as a first‐line ET, menopausal status, and sites of disease metastasis), we corroborated our conclusion (36 months [95% CI: 20–40] vs. 26 months [95% CI: 21–38], *p* = .69, Figure 3B).

**FIGURE 4:**
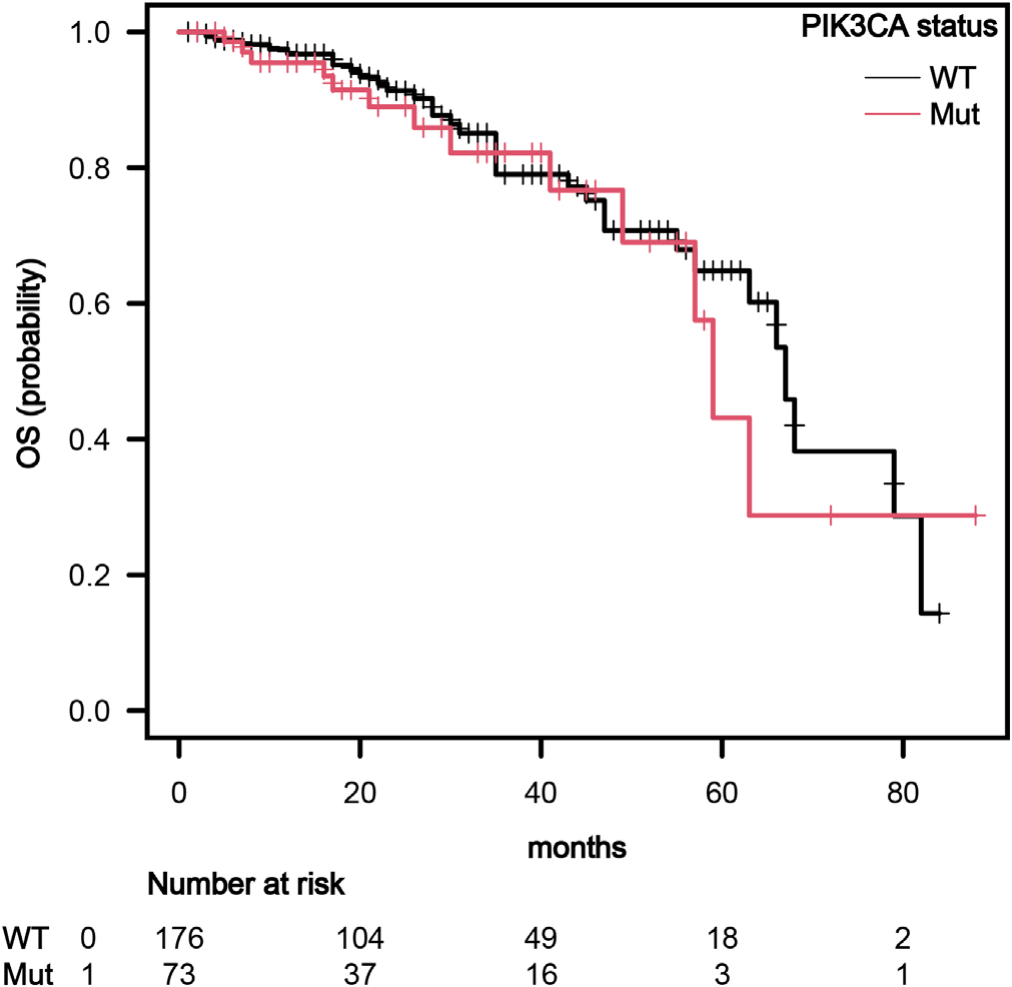
Kaplan‐Maier estimates of overall survival (OS). Patients with *PIK3CA* mutations did not have significantly different median OS compared to WT patients (59 months [95%, CI: 47–69] vs. 61 months [95%, CI: 55–67], [*p* = .60]).

A progression of the disease was detected in 26 patients <6 months at a first‐line endocrine therapy. The patients with *PIK3CA*muts with primary endocrine resistance were six (*N* = 6) (50% of which [*N* = 3] received CDK4/6 inhibitor plus ET). The patients with WT and primary endocrine resistance were twenty (*N* = 20) (35% of which [*N* = 7] received CDK4/6 inhibitor plus ET). The percentage of patients with primary endocrine resistance was not significantly different between the *PIK3CA*muts and WT cohorts, with 8.2% and 11.2% respectively (*p* = .47). The median OS for *PIK3CA*muts patients was not significantly different from those without mutation (59 months [95% CI: 47–69] vs. 61 months [95% CI: 55–67], *p* = .600, Figure 4).

## 4 DISCUSSION

The prevalence of *PIK3CA*mut in Bulgaria did not differ from that presented in TCGA as of January 4, 2023, and the existence of *PIK3CA* mutations may not affect clinical outcome. Our findings indicate also that the presence of *PIK3CA* mutations is not prevalent in patients with primary endocrine resistance.

In 30%–40% of HR+/HER2– BC patients, activating mutations were found in the *PIK3CA* gene, making it one of the most commonly altered genes in this type of tumor. The PI3K signaling pathway is a key regulator of cellular growth, metabolism, proliferation, survival, and invasion.^9^, ^13^ Constantly upregulated, the PI3K pathway is crucial for genomic stability preservation, chemoresistance, and cell survival, as it is involved in numerous DNA replication and cell cycle regulation processes. By reducing the activity of the spindle assembly checkpoint protein Aurora kinase B and, as a result, increasing the frequency of lagging chromosomes during prometaphase, PI3K inhibition may result in genomic instability and mitotic disastrous consequences.^14^

Several genetic changes have been identified in various tumor pathways, including deletion of *PTEN*, amplification of *AKT1* and *PIK3CA*, and somatic mutations in *PIK3CA* and *AKT1*.^9^, ^15^, ^16^ The majority of point mutations in the *PIK3CA* gene are located in the p110 cluster, specifically around two hotspots: E542/5 in the helical domain (exon 9) and H1047R near the catalytic domain (exon 20).^17^ These mutations lead to amino acid changes (E545K, E542K, and H1047R) that enhance the activity of the PI3K holoenzyme and result in constant AKT activity.^11^, ^18^ The activating *PIK3CA* mutations are present from the onset of oncogenesis in BC and are not typically lost or acquired during clonal development in the later latter phases of the disease, suggesting that they are driver mutations.^10^, ^11^, ^19^

Many PI3K‐inhibitors have been created in recent years, some of which have shown to be extremely toxic and inapplicable in clinical practice. SOLAR‐1 was a phase III, randomized, double‐blind, placebo‐controlled trial that investigated the effectiveness of the combination of fulvestrant and the α‐specific PI3K inhibitor alpelisib compared to placebo in patients with HR+, HER2–metastatic BC who had previously received treatment with aromatase inhibitors.^20^ In the group treated with alpelisib‐fulvestrant, the median PFS was 11.0 months, while in the placebo‐fulvestrant group, it was 5.7 months. However, the enhancement in OS was only numerical, not statistically significant.^18^, ^20^ Alpelisib has demonstrated efficacy with a tolerability profile that is manageable, however in the era of CDK4/6 inhibitors, it is still unclear which treatment modality should be delivered.

According to the current study, the prevalence of *PIK3CA*mut in Bulgarians is 29.2%, which is equivalent to the prevalence identified in several international studies. Anderson et al., 2020 conducted a systematic evaluation of 572 papers and conference abstracts to determine the prevalence of the *PIK3CA*mutation among HR+/HER2–metastatic BC. Based on the included literature, the median prevalence was 36.4%.^21^

We did not find a significant difference in the median PFS of first‐line ET ± CDK4/6 between patients with *PIK3CA* mutations and WT patients. Similar results were obtained in the OS analysis. According to the most recent study reports and meta‐analysis, patients in the WT cohort had a longer median PFS than patients in the *PIK3CA*mut cohort.^12^ In a study by Fillbrunn et al., 2022 the majority of patients got ET monotherapy or in combination with CDK4/6‐inhibitor or mTOR‐inhibitor, and more frequently as a second and subsequent line of therapy; this may explain why our results differ from those previously published. The discrepancy between our results and those of other studies may be due to the small number of patients in our study and the higher percentage of individuals with endocrine therapy resistance in the WT group.

Through the subgroup analysis of the MONARCH 2 research, it was found that the populations with *PIK3CA*mutations and the WT had comparable PFS and OS, indicating that the existence of the mutation probably did not play a role in the development of therapeutic resistance in patients who received CDK4/6‐inhibitor plus ET.^22^

According to a report by Ortega et al., 2020, the existence of *PIK3CA* mutations was not linked to CDK4/6 inhibitor resistance in terms of PFS. However, among patients who progressed on first‐line endocrine treatment in less than 6 months, the prevalence of *PIK3CA* mutations was higher (46.67%). These patients exhibited primary endocrine resistance.^23^ In our research, 26 individuals progressed less than six months of endocrine therapy, and we did not notice a statistically significant difference in the proportion of patients with primary endocrine resistance between *PIK3CA* mutated and WT patients. In addition, we noticed that the mutations were present in individuals with metastatic illness at the time of diagnosis, suggesting that the existence of *PIK3CA*muts is indicative of a more aggressive disease in these patients.

Similar to other retrospective real‐world evidence studies, this one is subject to some limitations, which should be taken into account when interpreting the results. Retrospective, nonrandomized investigations of the real world are susceptible to bias and confounding. Only physicians willing to participate in data collection were enrolled in the trial, which carries the possibility of selection bias. Other limitation is that the cause of death was not specified in calculating PFS and 15 patients underwent chemotherapy as a first line of treatment without any information regarding its duration or effects. An additional limitation is that our investigation was limited to the *PIK3CA* mutation with no available data on other prevalent genetic alterations such as TP53.

Not with standing these limitations, our real‐world trial offers some guidance on the timing of the introduction of novel PI3K inhibitors into the therapy regimen for patients with metastatic hormone‐positive HER2–disease. In conclusion, our research offers an important understanding of the subject, however, due to constraints such as its retrospective nature and limited number of participants, the results should be confirmed by more rigorous studies with larger sample sizes before more conclusive statements can be made.

## AUTHOR CONTRIBUTIONS

**R. Gencheva:** Investigation (equal); resources (equal); visualization (equal); writing – original draft (equal); writing – review and editing (equal). **M. Petrova:** Conceptualization (equal); investigation (equal); resources (equal); writing – original draft (equal). **P. Kraleva:** Investigation (equal); resources (equal); writing – original draft (equal). **S. Hadjidekova:** Methodology (equal). **M. Radanova:** Investigation (equal); visualization (equal). **N. Conev:** Investigation (equal); resources (equal). **D. Stoyanov:** Investigation (equal). **J. Arabadjiev:** Investigation (equal). **E. Tazimova:** Investigation (equal). **S. Bachurska:** Methodology (equal). **M. Eneva:** Resources (equal). **M. Tsvetkova:** Resources (equal). **G. Zhbantov:** Resources (equal). **T. Karanikolova:** Resources (equal). **D. Manov:** Resources (equal). **A. Ivanova:** Resources (equal). **M. Taushanova‐Hadjieva:** Resources (equal). **R. Staneva:** Methodology (equal). **E. Dimitrova:** Supervision (equal); writing – review and editing (equal). **I. Donev:** Formal analysis (equal); supervision (equal); visualization (equal); writing – review and editing (equal).

## CONFLICT OF INTEREST STATEMENT

M. Tsvetkova is an employee of Novartis Bulgaria. Dr. R. Gencheva, Prof. S. Hadjidekova, Assoc. Prof. N. Conev, Dr. D. Stoyanov, Asoc. Prof. J. Arabadjiev, Dr. E. Tazimova, Assoc. Prof. S. Bachurska and Dr. G. Zhbantov report a relationship with Novartis Bulgaria or Bulgarian Association of Medical Oncology that includes: speaking and lecture fees. The other authors declare no conflict of interest.

## ETHICS STATEMENT

The study received approval from the hospital's Scientific Research Ethics Committee and all participants have provided informed written consent. (Version 1/02.Apr.2021).

## Data Availability

The data that support the findings of this study are available from the corresponding author upon reasonable request.
